# Diagnosis and treatment of secondary nephrotic syndrome with rash as the first symptom: a case report

**DOI:** 10.1186/s12882-024-03665-0

**Published:** 2024-07-15

**Authors:** Bowen Qin, Yueqiang Li, Dong Kuang, Xi Yang, Chunyu Pan, Xiaojing Cai, Junhua Li

**Affiliations:** 1 Department of Nephrology, Tianyou Hospital, Wuhan University of Science and Technology, Wuhan, China; 2 Department of Nephrology, Tongji Hospital, Tongji Medical College, Huazhong University of Science and Technology, 1095 Jiefang Ave, Wuhan, 430030 China; 3 Department of Pathology, Tongji Hospital, Tongji Medical College, Huazhong University of Science and Technology, Wuhan, China

**Keywords:** Secondary membranous nephropathy, Mantle cell lymphoma, Non-hodgkin lymphoma, Rash, Case report

## Abstract

**Background:**

Membranous nephropathy (MN) is a common type of nephrotic syndrome (NS) in adults, accounting for about 20–30% of cases. Although secondary to specific factors, the coexistence of MN and mantle cell lymphoma (MCL) has been scarcely reported in clinical literature.

**Case presentation:**

A 59-year-old Chinese male was admitted to the hospital with a generalized pruritic rash with bilateral lower extremity edema, which did not improve significantly after symptomatic treatment. He had undergone renal biopsy, and the diagnosis was thought to be secondary MN (SMN), therefore, we did a lymph node biopsy on the patient and found that MN was complicated with MCL. Soon after, the patient was admitted to the hematology department for a BR chemotherapy regimen (composed of bendamustine 90 mg/m^2^ BSA (body surface area), rituximab 375 mg/m^2^ BSA and dexamethasone 5 mg), and during the post-treatment follow-up, both his symptoms and renal function improved.

**Conclusions:**

The mechanism underlying the combination of SMN and MCL remains elusive and exceedingly rare, consequently often overlooked in clinical practice. This case serves to offer valuable clinical insights for diagnosis and treatment, while emphasizing the pivotal role of renal pathology in clinical assessment.

## Background

NS is a group of clinical manifestations characterized by massive proteinuria (> 3.5 g/d) with hypoalbuminemia (< 30 g/L), edema, and hyperlipidemia, as well as other complications. Numerous conditions, including infection, secretory metabolism, autoimmune disease, cancer, etc., might have a secondary effect on NS. Renal biopsy and immunohistochemistry are required for patients with NS of unclear cause when necessary. This article describes a rare instance of NS secondary to MCL with a rash as the initial sign; following chemotherapy, the patient’s renal function recovered.

## Case presentation

In 2019, a 59-year-old man developed a generalized pruritic rash after being bitten by mosquitoes. He was hospitalized to our hospital in 2021 after receiving treatment with Chinese traditional medicine and angiotensin receptor blockers (ARBs), which did not significantly reduce proteinuria, and a urine analysis revealed urinary protein 3 + with bilateral lower extremities edema.

Serum creatinine (Scr) of 107 µmol/L, estimated glomerular filtration rate (eGFR) of 65.1 ml/min/1.73m^2^, albumin (ALB) of 32.3 g/L, urinary albumin-to-creatinine ratio (UACR) of 457.9 µg/mg, alkaline phosphatase (ALP) of 199 U/L, and lactate dehydrogenase (LDH) of 244 U/L were the laboratory results at the time of admission. Urinalysis showed protein 3+, erythrocytes 3+, and leukocytes 1+. Anti-neutrophil cytoplasmic antibodies (ANCAs), anti-nuclear antibodies (ANAs), anti-phospholipase A2 receptor (PLA2R) antibodies, anti-glomerular basement membrane (GBM) antibodies, serum M-protein, hepatitis B antibodies, hepatitis C antibodies, and syphilis spirochete antibodies were all negative.

Upon physical examination, there was minor edema in both lower extremities, a widely dispersed brown rash on the skin of the extremities and trunk, and slightly enlarged superficial lymph nodes palpable in the neck and axillae bilaterally.

### Skin performance

**Fig. 1:**
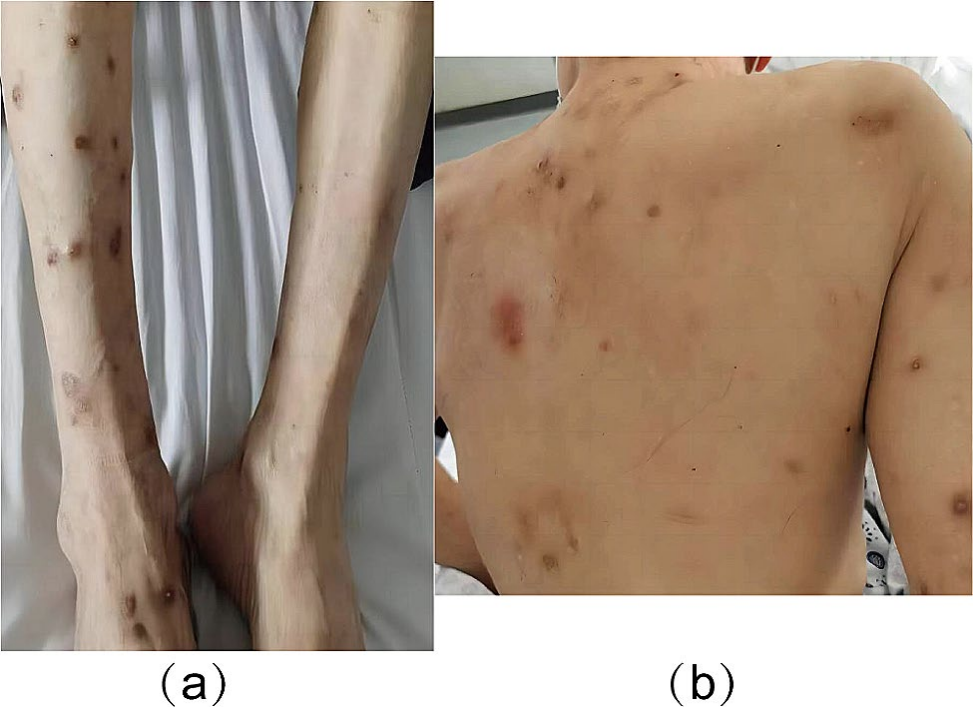
Scattered rash all over the body with crusting. Brown nodules on the limbs and trunk

Broad brown rash the size of a bean that is widely distributed across the trunk and limbs, especially the extremities, with nodules and discolored patches. There is a noticeable itching sensation, scrapes, and some cracked and crusted nodules along with the rash. (Fig. 1).

### Renal histology

**Fig. 2:**
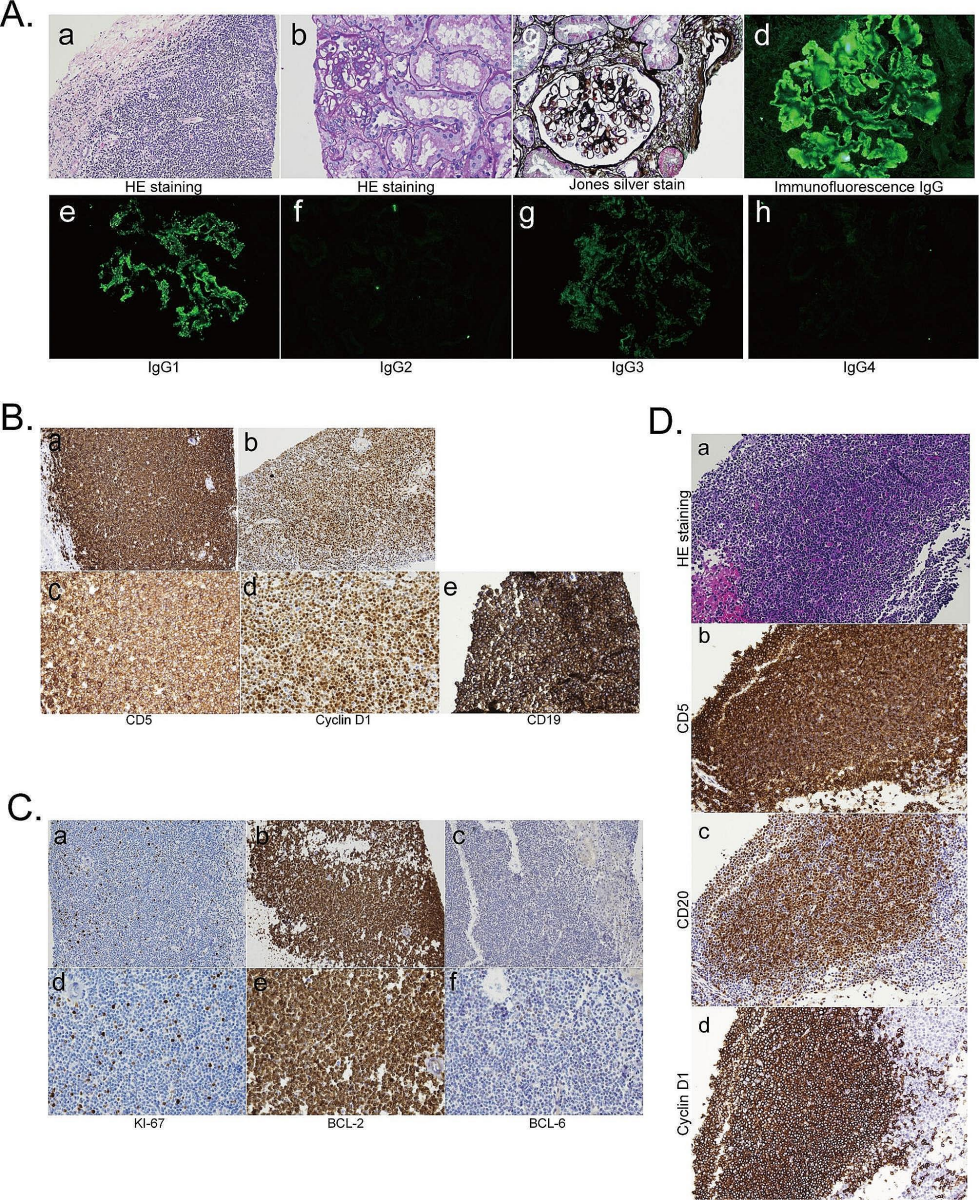
**A**. Light microscopy and IF of renal biopsies in SMN. (a) Mononuclear lymphocytes, plasma cells with large patchy and multifocal infiltration, and scattered eosinophilic infiltration were seen in the renal interstitium. (hematoxylin-eosin staining, Original magnification, ×200). (b) Mild segmental hyperplasia of glomerular mesangial cells and basement membranes. (hematoxylin-eosin staining, Original magnification, ×400). (c) Diffuse thickening of the glomerular basement membrane, with “spikes” formation. (Jones silver stain, Original magnification, ×400). (d) IF reveals diffuse granular staining along the GBM for IgG. (Original magnification, ×400). (e、f、g and h) IgG1 and IgG3 were positive, and IgG2 and IgG4 were not detected. **B**. IHC of infiltrating cells in MCL. (a and c) CD5 was positively expressed in a focal distribution under high magnification. (Original magnification, a ×200, c ×400). (b and d) Interstitial infiltrate positive staining for cyclin D1. (Original magnification, b ×200, d ×400). (e) Renal interstitium CD19 positive with diffuse distribution. (Original magnification, ×400). **C**. IHC of infiltrating cells in MCL. (a and c) CD5 was positively expressed in a focal distribution under high magnification. (Original magnification, a ×200, c ×400). (b and d) Interstitial infiltrate positive staining for cyclin D1. (Original magnification, b ×200, d ×400). (e) Renal interstitium CD19 positive with diffuse distribution. (Original magnification, ×400). **D**. Light microscopy and IHC of Lymph node biopsy. (a) Massive infiltration of disorganized small lymphocytes in the interstitium. (hematoxylin-eosin staining, Original magnification, ×200). (b) CD5 expression in lymph node interstitium with diffuse distribution. (Original magnification, ×200). (c) CD20 positive with diffuse distribution. (Original magnification, ×200). (d) Interstitial infiltrate positive staining for cyclin D1. (Original magnification, ×200)

A renal biopsy revealed sclerosis in one out of eight evaluated glomeruli. The remaining glomeruli exhibited mild proliferation of glomerular mesangial and stromal cells, diffuse thickening of the basement membrane with visible spike formation, significant inflammatory cell infiltration in the renal interstitium, and intimal thickening of small arteries. Eosinophilic deposits were observed in several areas. Immunofluorescence (IF) staining was specific for IgG, IgM, C3, and C1q, with IgG1 and IgG3 showing positive results. These granular deposits were positive for both λ-type and κ-type light chains, with no glomerular deposits of IgA, C4, FRA, ALB, PLA2R, or THSD7A detected. Electron microscopy revealed numerous electron-dense materials beneath the epithelial cells and within the basement membrane, along with diffuse podocyte process fusion (Fig. 2A). Immunohistochemistry (IHC) was positive for CD19, Cyclin D1, CD5, BCL2, and Ki-67 (Fig. 2B and C).

### Lymph node histology

MCL was identified by the massive infiltration of tiny cells with positive expression of CD5, CD19, CD20, CyclinD1, and PAX5. (Fig. 2D).

The patient’s urine output (UO) dropped to 800 ml/d between the first and third weeks following hospitalization, while the UACR rose to 953.6 µg/mg and the ALB level dramatically dropped. Using the results of renal biopsies and IHC, we determined that the patient had SMN in order to explain the reason for the patient’s ongoing proteinuria. The patient received a definitive diagnosis of MCL through lymph node biopsy. Bone marrow smear revealed abnormal lymphocytes in 10% and flow cytometry analysis of the bone marrow showed about 5.4% nucleated cells considered as abnormal mature B lymphocytes.

From the third week, he started chemotherapy with the BR regimen. For a total of twelve weeks, treatments were administered every four weeks. His kidney and liver function indices did not significantly change following the first round of chemotherapy, but there was a decrease in urine protein.

**Fig. 3:**
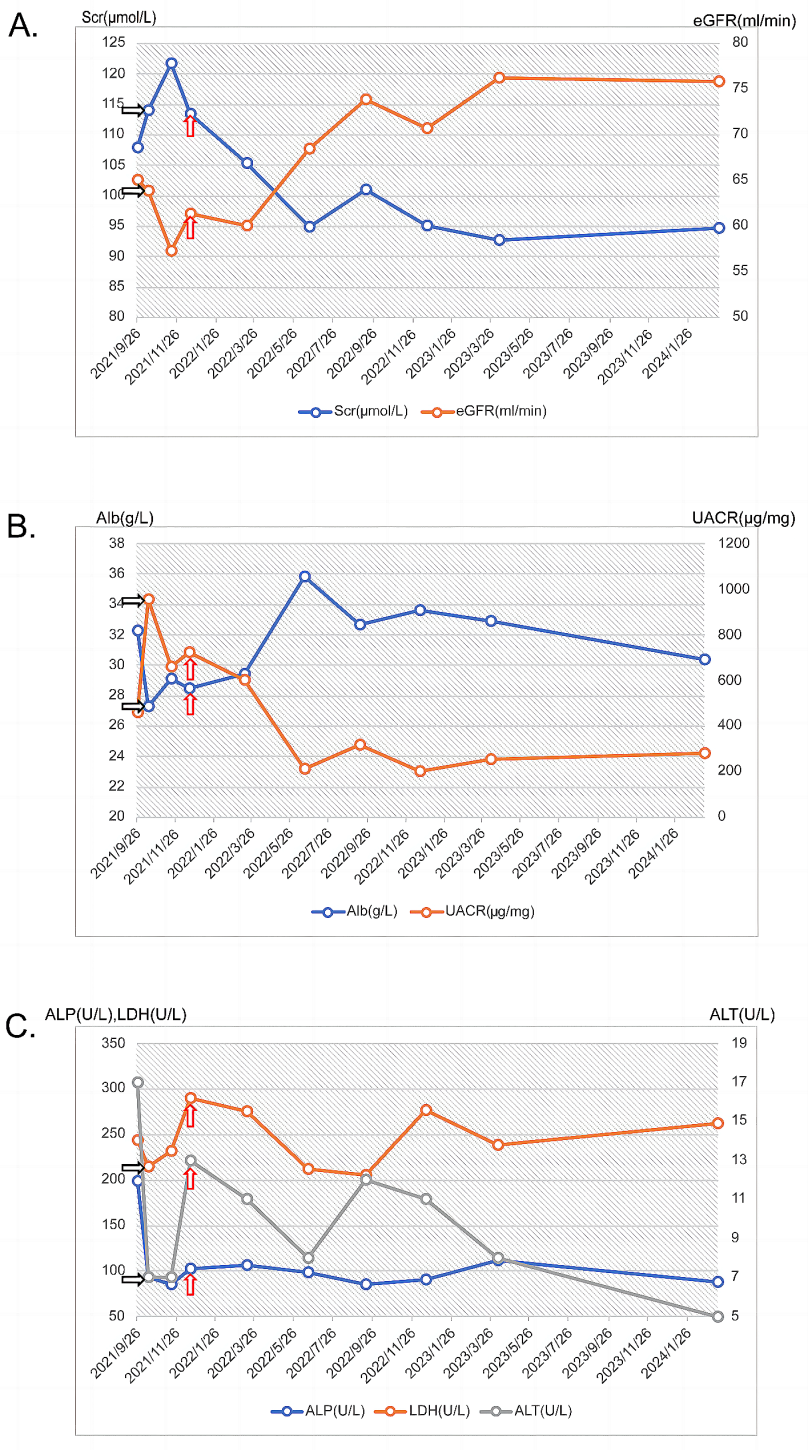
Changes in clinical indicators over time. Note: The patient’s initial chemotherapy treatment is indicated by the black arrow, and the chemotherapy treatment’s conclusion is indicated by the red arrow

The patient discontinued chemotherapy in the eleventh week after finishing the third course, and no severe side effects were noted during the course of treatment. Despite the fact that his renal function parameters were similar to those at admission due to hypoalbuminemia, his UACR dropped to an all-time low of 397.4 ug/mg and stayed stable after that. The patient had routine check-ups, and his kidney and liver functions improved. He also had normal white blood cell, platelet, and hemoglobin levels, and normal UO with considerably less foamy pee. His Scr level was 94.6 µmol/L, eGFR was 75.8 ml/min/1.73m^2^, UACR was 283.6 µg/mg, and ALB level was 30.4 g/L at the time of the most recent follow-up. (Fig. 3).

## Discussion

Most frequently, extranodal invasion of MCL is observed in the liver, colon, and stomach. Skin involvement in MCL is usually seen in 2–6% of patients with MCL [1]. The patient in this case initially presented with a generalized rash and then began to show signs of renal impairment, such as proteinuria and edema. When proteinuria did not improve after receiving treatment with ARBs and other drugs, a kidney biopsy was carried out to determine the cause. Hence, SMN was taken into account. Microscopically, we saw a significant infiltration of lymphocytes and plasma cells in the renal interstitium, which pointed us in the direction of hematologic disorders diagnosis. While it is uncommon for MN to coexist with hematologic malignancies, the patient’s renal IHC results led us to biopsy his lymph nodes, which further supported our concerns.

NS with a rash as the primary symptom may be secondary to other disorders in addition to this one. A patient with a scattered nonpruritic rash across the arms and back, followed by bilateral lower extremity depressed edema, has been documented and diagnosed with stage 2 syphilis with syphilitic MN based on the history of the rash, serology, and renal biopsy findings [2].

Treatment of the underlying illness is crucial, regardless of the initial symptom. Chemoimmunotherapy is still the preferred first-line treatment for MCL. Currently accepted treatment options include rituximab in combination with cytarabine as induction chemotherapy followed by rituximab in combination with autologous stem cell transplantation for maintenance therapy, in addition to a range of chemoimmunotherapeutic regimens such as R-CHOP, VR-CAP, and BR [3]. Apart from these therapies mentioned above, newer approaches to treating MCL have demonstrated encouraging outcomes: cellular therapies like chimeric antigen receptor (CAR) T cells and bispecific T-cell splicer (BiTe) antibodies; targeted medicines like BTK and BCL2 inhibitors [4].

A retrospective study collected all previous cases of MCL with abnormal renal function and found that all patients had improved or normalised renal function after lymphoma treatment [5]. In this instance, the patient received a BR regimen and saw a successful recovery of renal function following therapy. In comparison to rituximab combination chemotherapy, BR regimen has a lower toxicity profile, better toxicity tolerance, fewer side effects, and a higher safety profile when treating MCL or inactive B-cell non-Hodgkin’s lymphoma (NHL) [6, 7].

What is more worthy of our attention is that the suggestive role of IF and IHC in renal biopsy should not be underestimated. Among them, anti-PLA2R antibody and type 1 platelet reactive protein containing the 7 A structural domain (THSD7A) have important diagnostic significance for MN; moreover, deposition of IgG4 mainly in the glomeruli indicates primary MN (PMN), whereas significant deposition of other IgG subclasses may suggest SMN [8–10]. The absence of glomerular IgG4 deposits in patients with MN may be indicative of malignancy, and thus, IgG4 may also serve as a contrasting predictor of malignant tumorigenesis. SMN is usually caused by solid tumors, not by hematological malignancies, which are rare. In hematologic malignancies, renal injury associated with MCL usually also contains renal deposition of C3 or polyclonal immune deposits [5, 11].

In this instance, IF results for IgG4, PLA2R, and THSD7A in the kidneys were negative, indicating a predilection for SMN and the potential for a tumor. The patient’s kidney IHC results revealed positive expression of CD5, CD19, and CYCLIND1, which satisfies the most recent MCL diagnostic criteria. Interestingly, this patient demonstrated satisfactory survival during follow-up, even though BCL-2(+) and BCL-6(-) suggested a possibly unfavorable prognosis.

MCL can not only complicate NS but also coexist with a series of other renal diseases, such as membranoproliferative glomerulonephritis (MPGN), acute kidney injury (AKI), and lupus nephritis (LN) [12, 13]. Therefore, it is also necessary to screen for MCL or other hematologic disorders when making the corresponding renal disease diagnosis, and the mechanisms regarding the complications of these renal diseases remain to be explored.

## Conclusions

Finding the root cause of NS patients’ conditions is crucial when conservative treatment is unable to improve their state. Renal biopsy is still feasible and necessary for patients with renal insufficiency with a long clinical course, provided that the indications are met. Renal IHC plays a crucial role in the diagnosis of NS of unknown etiology. Our patient was diagnosed with MCL combined with MN by renal biopsy and IHC; following treatment under the BR regimen, his renal function improved. It is important to reflect that the mechanism of MN secondary to MCL still needs to be further explored clinically.

## Data Availability

No datasets were generated or analysed during the current study.

## Abbreviations

NS: Nephrotic syndrome
MN: Membranous nephropathy
MCL: Mantle cell lymphoma
SMN: Secondary membranous nephropathy
ARBs: Angiotensin receptor blockers
Scr: Serum creatinine
eGFR: Estimated glomerular filtration rate
ALB: Albumin
UACR: Urinary albumin-to-creatinine ratio
ALP: Alkaline phosphatase
LDH: Lactate dehydrogenase
ANCAs: Anti-neutrophil cytoplasmic antibodies
ANAs: Anti-nuclear antibodies
PLA2R: Phospholipase A2 receptor
GBM: Glomerular basement membrane
IF: Immunofluorescence
IHC: Immunohistochemistry
UO: Urine outpu
CAR: Chimeric antigen receptor
BiTe: Bispecific T-cell splicer
NHL: Non-Hodgkin’s lymphoma
THSD7A: The 7 A structural domain
PMN: Primary membranous nephropathy
MPGN: Membranoproliferative glomerulonephritis
AKI: Acute kidney injury
LN: Lupus nephritis
